# Relapse Rates and Predictors for Relapse in Ulcerative Colitis and Crohn’s Disease Patients After Discontinuation of Vedolizumab or Ustekinumab: The REVEUS Study

**DOI:** 10.3390/jcm14061793

**Published:** 2025-03-07

**Authors:** Alessandro Massano, Edoardo Vincenzo Savarino, Simone Saibeni, Cristina Bezzio, Lorenzo Bertani, Gian Paolo Caviglia, Marta Vernero, Angelo Armandi, Davide Giuseppe Ribaldone

**Affiliations:** 1 Gastroenterology Unit, Department of Surgery, Oncology and Gastroenterology, Azienda Ospedale Università Padova, University of Padua, 35121 Padova, Italy; edoardo.savarino@unipd.it; 2 IBD Centre, Gastroenterology Unit, Rho Hospital, ASST Rhodense, 20017 Rho, Italy; saibo@tiscali.it; 3 IBD Centre, IRCCS Humanitas Research Hospital, 20089 Rozzano, Italy; cribezzio03@yahoo.it; 4 Department of Biomedical Sciences, Humanitas University, 20072 Pieve Emanuele, Italy; 5 Azienda Ospedaliera Universitaria Pisana, 56126 Pisa, Italy; lorenzobertani@gmail.com; 6 Department of Medical Sciences, University of Turin, 10126 Turin, Italy; gianpaolocaviglia@unito.it (G.P.C.); marta.vernero@unito.it (M.V.); angelo.armandi@unito.it (A.A.); davidegiuseppe.ribaldone@unito.it (D.G.R.)

**Keywords:** biologics, anti-integrin, anti-IL12/23, target therapy, relapse, predictors, discontinuation, withdrawal, retreatment, advanced therapy

## Abstract

**Background/Objectives**: In the current era of tailored therapy, biologics such as vedolizumab (VDZ) and ustekinumab (UST) are increasingly administered to inflammatory bowel disease (IBD) patients. The decision to discontinue biologics after side effects or a lack of response is usually simple, but the decision to stop treatment in patients in remission is more difficult: to date, no study has been conducted to investigate the effects of VDZ or UST withdrawal. Our study aims to investigate the rates and predictors of relapse of IBD after the discontinuation of VDZ and UST during a well-controlled disease phase and to evaluate the response to retreatment. **Methods**: In this observational, multicenter, retrospective study, we included IBD patients who discontinued VDZ or UST during a well-controlled disease phase after at least 1 year of treatment. We collected demographic and clinical data for each patient at the time of discontinuation and at follow-up visits. **Results**: We included 36 IBD patients from 5 different centers; 80.0%, 58.5%, and 48.3% of patients maintained clinical remission at 12, 24, and 48 months after discontinuation, respectively. Crohn’s disease (CD) patients were more likely to maintain remission than ulcerative colitis (UC) patients at 48 months (70.0% vs. 40.0%). No predictors of relapse were identified, but UC patients had a higher risk of early relapse than CD patients (HR = 3.23); 81.3% of retreated IBD patients achieved clinical remission after induction and at 12 months. **Conclusions**: No predictors of disease relapse after treatment discontinuation were identified. Half of the patients had a relapse within 48 months after discontinuation, but most of them achieved clinical remission after retreatment.

## 1. Introduction

Inflammatory bowel disease (IBD) is a group of chronic, idiopathic, and multifactorial diseases of the gastrointestinal tract, including Crohn’s disease (CD) and ulcerative colitis (UC) [1]. Genetic and environmental factors, gut dysbiosis, impaired intestinal permeability and inflammatory and immunological dysregulation are involved in the etiopathogenesis of the disease [2].

IBD is characterized by an abnormal immunological response to different endogenous and exogenous factors, resulting in a shift towards pro-inflammatory pathways that cause chronic inflammation of the gastrointestinal tract [3]. In the last decades, the increasingly deeper knowledge of the pathogenesis of the disease led to the development of advanced therapies, such as biologics and small molecules, that target and inhibit specific molecules involved in the inflammatory response [4].

To date, biologics cannot cure IBD, but they have been shown to effectively induce and maintain clinical remission and promote mucosal healing in a significant percentage of IBD patients [5,6,7,8]. Vedolizumab (VDZ) and ustekinumab (UST) are biologic agents that block the activity of integrin α4β7 and both interleukin 12 (IL-12) and IL-23, respectively [9]. Patients who achieve remission continue to be treated with biologics for many years because of their efficacy and safety in keeping long-term remission [10,11,12,13].

The decision to stop biologics—including VDZ or UST—for primary non-response, secondary loss of response, or severe adverse events (AEs) is usually simple and rational, but there is no clear guidance on the appropriate duration of biologic treatment or the optimal time for its discontinuation in patients in remission [14,15]. This decision is influenced by factors such as doctor and patient preference, treatment duration, treatment adherence, and costs [16,17,18].

Although there are existing studies on the discontinuation of anti-TNFα after achieving remission, only one study evaluated the effect of VDZ withdrawal and retreatment, and no study has been conducted to investigate these aspects for UST [19,20,21,22,23,24,25,26,27].

Specifically, elective discontinuation of VDZ and UST is of particular interest for several reasons: the increasing number of patients on biologic therapy contributes to rising healthcare costs; adherence to long-term treatment is generally low, often resulting in suboptimal treatment outcomes; and although it is effective and safe, long-term treatment is not completely free from AEs [18,28].

In this multicenter, retrospective, observational study, we aim to evaluate relapse rates of IBD after the discontinuation of VDZ or UST during remission or mild clinical activity, predictive factors associated with relapse, and response to retreatment. These real-life findings might be useful for the development of future prediction models and guidelines.

## 2. Materials and Methods

### 2.1. Study Design and Data Collection

We conducted an observational, multicenter, retrospective study in 5 different centers: A. O. U. Città della salute e della Scienza di Torino (Turin, Italy), Azienda Ospedale—Università Padova (Padua, Italy), Rho Hospital (Rho, Italy), IRCCS Humanitas Research Hospital (Rozzano, Italy), and University Hospital of Pisa (Pontedera, Italy).

This study followed the principles of the Declaration of Helsinki and was approved by the local ethical committees: Comitato Etico Interaziendale A.O.U. Città della Salute e della Scienza di Torino—A.O. Ordine Mauriziano—A.S.L. Città di Torino (protocol code 0109499 12 November 2020).

We included patients that met all of the following inclusion criteria:

1. ≥18-yearold male and female patients with a confirmed diagnosis of IBD according to ECCO-ESGAR Guidelines [29].
2. Patients who discontinued treatment with VDZ or UST during clinical remission or mild clinical activity, after being treated for at least 1 year, up to March 2023.
3. Reasons for discontinuation included physician’s choice, patient’s preference, pregnancy, low adherence to therapy, or safety issues.
4. Maintenance therapy after discontinuation was mesalamine or no drug.

The exclusion criteria were as follows:

1. Patients with moderate or severe clinical or endoscopic activity of disease when VDZ or UST was discontinued.
2. Patients who were treated with another biologic, immunosuppressant, or corticosteroids immediately after VDZ or UST discontinuation.
3. Patients with total colectomy in past medical history.

For all included patients, we performed a retrospective collection of hospital records. We collected data about age, gender, IBD type, date of diagnosis, smoking habits, target therapy start and stop date, treatment schedule, duration of treatment, disease duration, laboratory results—C reactive protein (CRP) and fecal calprotectin—Montreal classification, Harvey-Bradshaw Index (HBI), Simple Endoscopic score for Crohn’s disease (SES-CD), partial Mayo Score (pMayo), Mayo Endoscopic Score (MES), reasons for discontinuation, and histological activity.

All included patients were prospectively followed at the outpatient clinics by IBD experts with regular appointments from the time of target therapy discontinuation (baseline) until the last follow-up visit. Clinical, biochemical, and endoscopic evaluations were performed during follow-up every 6 months.

Clinical remission was defined as an HBI ≤ 4 for patients with CD and a pMayo ≤ 1 for patients with UC, without any concurrent oral corticosteroid therapy; mild clinical activity was defined as an HBI of 5–7 for patients with CD and a pMayo 2–4 for patients with UC, without any concurrent oral corticosteroid therapy [30,31]. We excluded patients with moderate to severe clinical activity at baseline (i.e., pMayo ≥ 2 or HBI ≥ 8).

We defined endoscopic remission as SES-CD = 0–2 for CD, and as MES = 0 for UC; mild endoscopic activity was defined as SES-CD = 3–6 for CD, and MES = 1 for UC. We excluded patients with moderate to severe endoscopic activity at baseline (i.e., MES ≥ 2 or SES-CD ≥ 7).

The duration of follow-up and occurrence of relapse were documented. A relapse was defined as the requirement for retreatment with a target therapy due to IBD flare. In case of relapse, treatment decision was made at physician’s discretion. In case of retreatment, we documented if the retreatment turned out successful.

Response to retreatment with target therapy was defined as a pMayo ≤ 2 for patients with UC and as an HBI ≤ 4 for patients with CD without oral corticosteroid and with target therapy not discontinued at the end of follow-up post relapse.

We included patients who discontinued VDZ or UST up to March 2023 to have at least 1 year of follow-up after discontinuation. For the same reason, if a patient had a relapse, we included the patient only if the relapse happened up to March 2023 to have at least 1 year of follow-up after retreatment.

### 2.2. Statistical Analysis

Descriptive statistics were used to characterize the patient population. Results were provided as numbers (percentages) for discrete variables and median (range) for continuous variables and as frequencies and percentages for categorical variables. Paired parameters were tested with the Wilcoxon signed-rank test. Time to relapse and relapse rates were assessed using the Kaplan–Meier analysis. Univariable Cox regression analysis was used to study potential predictors for relapse. Multivariable Cox proportional hazard analysis was performed for proportional hazard assumption. A *p*-value of <0.05 was considered statistically significant.

All statistical analyses were performed using MedCalc Statistical Software version 18.9.1 (MedCalc Software bvba, Ostend, Belgium; http://www.medcalc.org; 2018).

## 3. Results

### Population Characteristics

We included 36 IBD patients who discontinued VDZ or UST during a clinical remission or mild clinical activity: among them, 29 patients (80.6%) discontinued VDZ, while 9 patients (19.4%) discontinued UST.

In our study population, 24 patients (66.7%) had UC, and 12 patients (33.3%) had CD.

Among the groups who discontinued the biologic drugs, the IBD type equally distributed (VDZ: 19 UC patients (65.5%) and 10 CD patients (34.5%); UST: 5 UC patients (71.4%) and 2 CD patients (28.6%); *p* = 0.7691).

The median age at baseline was 42.5 years old, and the group had a similar proportion of males and females: 17 patients (47.2%) are male, 19 patients (52.8%) are female.

At baseline, 11 patients (30.6%) were smokers, 4 patients (11.1%) were ex-smokers, and 21 patients (58.3%) were non-smokers.

Moreover, the median duration of treatment with VDZ or UST was 30 months, and the median disease duration was 120.5 months. Median CRP value was 2.0 mg/L and median fecal calprotectin value was 71.0 μg/g.

Before starting VDZ or UST, 30 patients (83.3%) had undergone one or more previous advanced therapies (30 anti-TNFα, 5 VDZ, 2 UST, 3 Janus kinase inhibitors (JAKi)). Among them, 12 patients (40%) discontinued the previous advanced therapy for primary failure, 14 patients (46.7%) for secondary loss of response, and 4 patients for intolerance (13.3%). Only 6 patients (16.7%) did not receive any advanced therapy before VDZ or UST.

Among all patients, 50.0% discontinued treatment on physician’s choice, while 50.0% discontinued it because of pregnancy, low adherence to therapy, or safety issues (1 of these patients discontinued treatment because he required liver transplantation).

In the CD group, 2 patients (16.6%) had mild clinical activity at baseline, while 10 patients (83.4%) were on clinical remission, based on HBI; 2 patients (20.0%) had mild endoscopic activity, and 8 patients (80.0%) were on endoscopic remission, based on SES-CD. In the UC group, 9 patients (37.5%) had mild clinical activity at baseline, while 15 patients (62.5%) were on clinical remission, based on pMayo; 7 patients (31.8%) had mild endoscopic activity, and 15 patients (68.2%) were on endoscopic remission, based on MES. Among all, only 3 patients (14.3%) had histological remission (absence of neutrophils on colonic biopsies): 2 of them had UC and 1 patient had CD.

Characteristics of the population at baseline are reported in Table 1.

**Figure 1:**
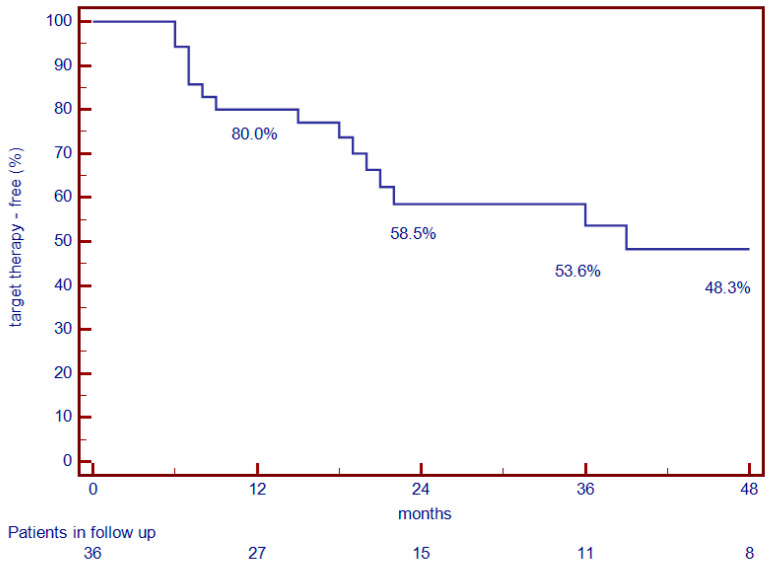
Percentages of IBD patients maintaining target-therapy-free remission.

**Figure 2:**
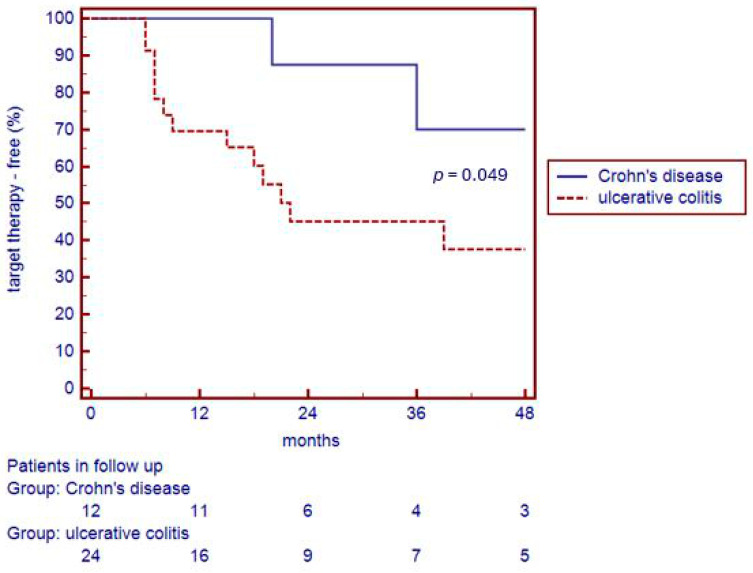
Percentages of CD and UC patients maintaining target-therapy-free remission.

Throughout the entire follow-up period (median duration 20.0 months), half of the patients (50.0%) had an IBD relapse requiring a retreatment with a target therapy. Relapses increased over time after the discontinuation of biologics: in fact, 80.0% of IBD patients maintained biologic-free remission after 12 months, 58.5% after 24 months, 53.6% after 36 months, and only 48.3% after 48 months (Figure 1). Instead, considering the two groups separately, 100% of CD patients maintained biologic-free remission after 12 months, 85.0% after 24 months, and 70.0% after 48 months, while 70.0% of UC patients maintained biologic-free remission after 12 months, 45.0% after 24 months, and 40.0% after 48 months (Figure 2). The Kaplan–Meier analysis revealed a significant difference in biologic-free remission between IBD types, with the UC patients having higher relapse rates compared to CD patients (log-rank *p* = 0.049).

Since the Kaplan–Meier survival analysis showed a statistically significant difference between CD and UC patients, we further analyzed baseline characteristics among CD and UC patients to identify possible confounding factors (Table 2). This analysis revealed that disease duration and CRP values at baseline were different among the two groups, with higher CRP values and longer disease duration in CD patients (*p* = 0.020; and *p* = 0.002, respectively).

A Cox regression analysis showed that biologic-free remission rates across different IBD types had no statistically significant difference when adjusted for CRP values and disease duration at baseline (*p* = 0.143).

We further analyzed the baseline characteristics of the patients to assess their ability to predict the need for a retreatment at univariate analysis. Sex, age, smoking habits, and IBD type were not able to predict the risk of relapse: for male sex, hazard ratio (HR) = 0.72, 95% Confidence Interval (CI) HR = 0.28–1.83, *p* = 0.49; for UC, HR = 3.22, 95% CI HR = 0.93–11.17, *p* = 0.06; for active smoking, HR = 0.90, 95% CI HR = 0.27–3.04, *p* = 0.88.

The risk of relapse was independent of the discontinued biologic (VDZ or UST) at baseline (HR = 0.49, 95% CI HR = 0.11–2.17, *p* = 0.35), the duration of treatment (HR = 3.22, 95% CI HR = 0.38–27.09, *p* = 0.28), or the previous escalation of treatment (HR = 1.03, 95% CI HR = 0.99–1.07, *p* = 0.12).

We did not find any association between the risk of relapse and CRP values (HR = 1.00, 95% CI HR = 0.88–1.13, *p* = 0.97), fecal calprotectin values (HR = 1.00, 95% CI HR = 0.99–1.00, *p* = 0.37), clinical activity of disease at baseline (HR = 0.57, 95% CI HR = 0.07–4.33, *p* = 0.58), endoscopic activity (HR = 0.44, 95% CI HR = 0.10–1.94, *p* = 0.28), or histological remission (HR = 1.21, 95% CI HR = 0.31–4.73, *p* = 0.77). All 3 patients with histological remission relapsed after treatment discontinuation during follow-up.

There was no statistical significance in the association between the reason of treatment discontinuation (such as physician’s decision for clinical remission, pregnancy, low adherence, or safety issues) and the need for a retreatment (HR = 0.80, 95% CI HR = 0.29–2.17, *p* = 0.66).

The results of univariate analysis are reported in Table 3.

Of the 18 patients who had a relapse and required a retreatment, 2 patients (11.1%) were retreated with infliximab (IFX), 5 patients (27.8%) with UST, and 9 patients (50.0%) with VDZ; guselkumab was used in 1 patient (5.6%) and risankizumab in 1 patient (5.6%). Retreatment was effective in 15 patients (83.3%), achieving steroid-free clinical remission after 12 months; it was ineffective in 3 patients (18.7%) only.

Among the 13 patients who discontinued VDZ at baseline and had a relapse, 9 patients (69.0%) were retreated with the same target therapy, achieving steroid-free clinical remission in 7 cases (88.0%). In the group of patients who discontinued UST at baseline and had a relapse, one patient was retreated with the same biologic drug, and he achieved steroid-free clinical remission after 12 months of retreatment.

## 4. Discussion

Our study aimed to analyze relapse rates and potential predictors of relapse in IBD patients following the discontinuation of VDZ or UST during clinical remission after being treated for at least 12 months.

In the literature, there is only one retrospective observational study that evaluated the rate of clinical remission maintenance in 95 IBD patients who discontinued VDZ during clinical remission. It was observed that 64.0% of patients experienced a relapse after a median follow-up of 11.2 months following VDZ discontinuation. Twenty-four of these patients were retreated with VDZ, and 71.0% of them benefited from the retreatment, achieving steroid-free clinical remission again. This study did not analyze the potential predictive factors that could help identify patients more likely to have a IBD relapse [24]. Regarding UST, no studies can be found in the literature analyzing relapse rates or potential predictors of relapse following drug discontinuation in IBD patients on clinical remission.

Conversely, there are already some existing studies about the discontinuation of anti-TNFα in IBD patients after achieving remission, so more is known about relapse rates and predictors of relapse for anti-TNFα: the incidence rate of relapse is about 20% per patient-year in IBD patients who discontinued anti-TNFα after clinical remission was achieved, and retreatment is effective and safe in about 80% of these patients [21,22]. Also, several studies identified some profiles of patients who may not benefit from the discontinuation because of a higher risk of relapse (younger age, previous extensive or complicated disease, persistent endoscopic lesions or elevated CRP or fecal calprotectin, etc.) and some characteristics that may favor the decision to discontinue the treatment (older age, normalization of CRP or fecal calprotectin, comorbidities, absence of residual trough level, low adherence, absence of drug reimbursement, etc.) [32].

Regarding sex, this research found no statistically significant differences between male and female groups in predicting the occurrence of relapse, as well as a multicenter study conducted by a Korean association for anti-TNFα drugs [20].

Regarding IBD type, analyzing both our subgroup of patients with UC (HR = 3.23, 95% CI 0.93–11.17, *p* = 0.06) and those from the GETAID-Vedo-STOP study with CD (HR = 1.26, 95% CI 0.74–2.14, *p* = 0.40), neither result is statistically significant [24]. However, in our study, there is a trend approaching statistical significance, suggesting that patients with UC are more likely to restart target therapy compared to those with CD. This is likely because UC is a much more symptomatic disease than CD, with a tendency to present clinical signs of disease reactivation earlier. This allows clinicians to recognize relapse more quickly and, consequently, restart treatment. In contrast, CD is a more insidious disease, with a smaller percentage of patients showing symptoms in the early phases of relapse, which delays treatment resumption.

Smoking habits were not associated with relapse, whereas a systematic review on anti-TNFα drugs identified smoking as a risk factor for IBD relapse (HR 1.91, 95% CI 1.11–3.27, *p* < 0.05) [33]. However, the results of our study align with those of a multicenter retrospective study that evaluated only UC patients, in which active smoking was not a statistically significant predictor of relapse after anti-TNFα stop (HR = 0.83, 95% CI 0.33–2.07, *p* = 0.69) [22].

In the GETAID-Vedo-STOP study, the patient’s choice to discontinue therapy was found to be a protective factor against the risk of relapse (HR = 0.40, 95% CI 0.20–0.76, *p* = 0.006) [24]. In our study, due to the low sample size for each reason for discontinuation, we decided to compare two groups: one in which treatment was discontinued by the physician because the disease was well controlled, and another that included other reasons (pregnancy, patient preference, low adherence, safety issues). The analysis of these two groups showed no statistically significant difference in the risk of relapse.

The optimization of the biologic agent before baseline was not found to be a predictive factor for relapse (HR = 3.22, 95% CI 0.38–27.09, *p* = 0.28). However, as described in the literature by Gisbert et al., the optimization of anti-TNFα therapy has been identified as a risk factor for relapse rate after treatment discontinuation [33].

The persistence of mild clinical disease activity at baseline was not found to be a predictor of relapse following the discontinuation of VDZ or UST (HR = 0.57, 95% CI 0.07–4.33, *p* = 0.58). Conversely, in the GETAID-Vedo-STOP study, the absence of clinical disease activity was identified as a protective factor (HR = 0.40, 95% CI 0.20–0.76, *p* = 0.006) [24].

Neither endoscopic activity nor histological activity at baseline were identified as predictors of relapse. However, it was observed that the discontinuation of anti-TNFα for CD on clinical remission was associated with a 42% relapse rate, while the relapse rate was lower when discontinued on endoscopic remission (26%) [33]. Similarly, in the GETAID-Vedo-STOP study, endoscopic remission was identified as a statistically significant protective factor (HR = 0.47, 95% CI 0.25–0.89, *p* = 0.02) [24].

Mucosal healing is likely a factor to consider when discontinuing anti-TNFα therapy, as patients in clinical, endoscopic, and histological remission seem to have a lower relapse rate. However, some studies report a high frequency of disease reactivation even in patients exhibiting these characteristics [33]. Similarly, in patients treated with VDZ and UST, these factors should be considered when deciding whether or not to discontinue therapy, although the exact degree of mucosal healing required is not yet fully understood [24,32].

Age at baseline does not appear to be a significant factor in predicting relapse (HR = 1.01, 95% CI 0.98–1.04, *p* = 0.56). This finding is consistent with a multicenter retrospective study that evaluated patients with UC on anti-TNFα therapy [22]. However, in another study and in the review of Gisbert et al., a younger age was associated with a higher risk of relapse, likely because younger patients tend to have a more aggressive disease course [21,33].

In our study, CRP and fecal calprotectin values were not statistically significant predictors of relapse (respectively, HR = 1.00, 95% CI 0.88–1.13, *p* = 0.97; HR 1.00, 95% CI 0.99–1.00, *p* = 0.37). In contrast, the GETAID-Vedo-STOP study found that a CRP value lower than 5 mg/L was a protective factor against the risk of relapse (HR = 0.53, 95% CI 0.31–0.90, *p* = 0.02) [24]. Similarly, for patients on anti-TNF therapy, low values of CRP and fecal calprotectin were associated with a lower risk of relapse [33].

Among all IBD patients, 20% required retreatment due to disease relapse after 12 months from discontinuation. After 24 months, this increased to 41.5%, and after 48 months, to 50%. Of these patients, 83.3% achieved steroid-free clinical remission. It is known from the literature that approximately 10–15% of patients who continue biologics despite remission will experience a relapse after 12 months of treatment, with this percentage rising to about 30% after 24 months [32,34]. These relapse rates are not so distant from those observed in our study among patients who discontinue treatment during remission, with a 5–10% difference at 12 months and approximately 20% at 24 months. This comes at the cost of significant expenses and potential side effects for patients who have not discontinued the target therapy. Furthermore, in patients who discontinue biologics, the vast majority of those who relapse regain clinical remission after retreatment. Therefore, utilizing a “therapeutic holiday” could be considered a potential strategy in the management of patients with IBD.

Our study has several limitations. First, the number of enrolled patients is relatively small, which may affect the statistical power of the analyses performed. Another limitation is the lack of a control group, which prevents the comparison of potential differences between a cohort of patients who discontinue biologics during remission and a cohort of patients who do not discontinue biologics. Identifying patients who discontinue the target therapy during remission is challenging, as treatment is typically continued until failure or loss of response or adverse events. Data collection was conducted retrospectively; therefore, some data may have been assessed inconsistently over time. Colonoscopies were performed by different operators and retrospectively reviewed using available reports, leading to heterogeneity in the reporting. The biopsies analyzed were not re-evaluated by the same pathologist, resulting in heterogeneous reporting. Finally, trough levels were not available.

This study has several strengths too. The enrolled patients, as they come from multiple centers, represent a heterogeneous sample, helping to avoid selection bias. Additionally, the follow-up of our patients is relatively long, allowing us to assess the medium- to long-term course of the disease. Finally, despite the limited sample size, this is the second study in the world regarding the discontinuation of VDZ during remission and the first ever to consider the discontinuation of UST. It is also the first to analyze the rate of retreatment after discontinuation of these biologics, following a treatment period of at least 12 months.

## 5. Conclusions

In conclusion, our study confirms that patients who discontinued VDZ or UST during remission had a significant rate of relapse leading to retreatment, with a 41.5% retreatment rate at 2 years after discontinuation.

We observed that UC patients tend to show early clinical signs of disease relapse compared to patients with CD (HR UC 3.23, 95% CI 0.93–11.17, *p* = 0.06).

Our analysis did not identify any statistically significant predictors of relapse, including sex; age; disease type; smoking habits; type; duration or escalation of biologics; clinical, endoscopic, or histological activity; and CRP or fecal calprotectin levels.

This result should be interpreted considering the limited sample size and the absence of a control group.

Expanding the sample size would be crucial to strengthen the validity of these findings. Ideally, a prospective study design with two cohorts—patients continuing vs. discontinuing treatment—would be necessary to achieve a more objective evaluation of relapse rates and predictors.

Regarding the success rate of retreatment after relapse, our data indicate that 83.3% of patients recapture disease remission after 1 year of retreatment, making a therapeutic vacation a feasible strategy, at least for a subset of patients.

## Figures and Tables

**Table 1:** Characteristics of the population at baseline.

Characteristics	Values
Age (years), median [IQR]	42.5 [29.0–53.5]
Sex (M/F), *n* (%)	17/19 (47.2%/52.8%)
Smoking habits (smokers/ex-smokers/non-smokers), *n* (%)	11/4/21 (30.6%/11.1%/58.3%)
Disease duration (months), median [IQR]	120.5 [58.5–177.0]
CRP (mg/L), median [IQR]	2.0 [0.9–3.2]
Fecal calprotectin (µg/dL), median [IQR]	71.0 [27.0–112.0]
IBD type (UC/CD), *n* (%)	24/12 (66.7%/33.3%)
Discontinued treatment (VDZ/UST), *n* (%)	29/7 (80.6%/19.4%)
Reason for discontinuation (physician’s choice/other), *n* (%)	18/18 (50.0%/50.0%)
IBD type in VDZ group (UC/CD), *n* (%)	19/10 (65.5%/34.5%)
IBD type in UST group (UC/CD), *n* (%)	5/2 (71.4%/28.6%)
Previous optimization of VDZ (yes/no), *n* (%)	2/27 (6.9%/93.1%)
Previous optimization of UST (yes/no), *n* (%)	0/7 (0.0%/100.0%)
Treatment duration (months), median [IQR]	30.0 [18.5–37.5]
Previous advanced therapy (yes/none), *n* (%)	30/6 (83.3%/16.7%)
Class of previous advanced therapy (anti-TNFα/VDZ/UST/JAKi), *n* (%)	30/5/2/3 (83.3%/13.9%/5.6%/8.3%)
Reason for previous advanced therapy discontinuation (primary failure/secondary non-response/intolerance), *n* (%)	12/14/4 (40.0%/46.7%/13.3%)
CD Montreal classification (L1/L2/L3/L4), *n* (%)	3/1/7/1 (25.0%/8.3%/58.3%/8.3%)
UC Montreal classification (E1/E2/E3), *n* (%)	2/13/9 (8.3%/54.2%/37.5%)
HBI (mild/remission), *n* (%)	2/10 (16.6%/83.4%)
pMayo (mild/ remission), *n* (%)	9/15 (37.5%/62.5%)
SES-CD for CD (mild/remission), *n* (%)	2/8 (20.0%/80.0%)
MES for UC (mild/remission), *n* (%)	7/15 (31.8%/68.2%)
Histological remission (yes/no), *n* (%)	3/18 (14.3%/85.7%)

CD: Crohn’s disease. CRP: C-reactive protein. F: female. HBI: Harvey-Bradshaw Index. IQR: interquartile range. JAKi: Janus kinase inhibitors. M: male. MES: Mayo Endoscopic Score. pMayo: partial Mayo Score. SES-CD: Simple Endoscopic score for Crohn’s disease. UC: ulcerative colitis. UST: ustekinumab. VDZ: vedolizumab.

**Table 2:** Baseline characteristics compared between IBD types.

Baseline Characteristics	UC	CD	*p* Value
Sex (M/F), n (%)	11/13 (45.8%/54.2%)	6/6 (50%/50%)	0.816
Smoking habits (smokers/ex-smokers/non-smokers), n (%)	5/3/16 (20.8%/12.5%/66.7%)	6/1/5 (50%/41.7%/8.3%)	0.201
Histological remission (yes/no), n (%)	2/12 (14.3%/85.7%)	1/6 (14.3%/85.7%)	1.000
Age (years), median [IQR]	36.0 [24.8–53.3]	47.5 [38.8–54.5]	0.202
Treatment duration with VDZ/UST (months), median [IQR]	30.0 [17.8–33.8]	30.0 [23.8–41.3]	0.383
CRP (mg/L), median [IQR]	1.0 [0.5–2.5]	2.8 [2–5.4]	0.020
Disease duration (months), median [IQR]	67.5 [52.8–139.8]	182.5 [144.0–284.8]	0.002
Fecal calprotectin (µg/dL), median [IQR]	72.5 [35.0–131.8]	63.5 [23.5–131.8]	1.000

CD: Crohn’s disease. CRP: C-reactive protein. F: female. IQR: interquartile range. M: male. UC: ulcerative colitis. UST: ustekinumab. VDZ: vedolizumab.

**Table 3:** Results of univariate analysis for potential predictors of relapse.

Variable	HR	95% CI HR	*p* Value
Male sex	0.72	0.28–1.84	0.49
UC	3.23	0.93–11.17	0.06
Smoking	0.91	0.27–3.04	0.88
UST	0.49	0.11–2.17	0.35
Physician’s choice	0.80	0.29–2.17	0.66
Previous optimization	3.22	0.38–27.09	0.28
Mild clinical activity	0.57	0.07–4.33	0.58
Endoscopic activity	0.44	0.10–1.94	0.28
Histological remission	1.22	0.31–4.73	0.77
Age	1.01	0.98–1.04	0.56
Treatment duration	1.03	0.99–1.07	0.12
CRP	1.00	0.88–1.13	0.97
Fecal calprotectin	1.00	0.99–1.00	0.37

CRP: C-reactive protein. HR: hazard ratio. UC: ulcerative colitis. UST: ustekinumab.

## Data Availability

The data presented in this study are available on request from the corresponding author.

## Abbreviations

The following abbreviations are used in this manuscript:

AEs	Adverse events
CD	Crohn’s disease
CRP	C-reactive protein
DOAJ	Directory of open access journals
HR	Hazard ratio
IBD	Inflammatory bowel disease
IL	Interleukin
IQR	Interquartile range
JAKi	JAK inhibitors
LD	Linear dichroism
MDPI	Multidisciplinary Digital Publishing Institute
MES	Mayo Endoscopic Score
pMayo	Partial Mayo Score
SES-CD	Simple Endoscopic score for Crohn’s disease
TLA	Three-letter acronym
TNF	Tumor necrosis factor
UC	Ulcerative colitis
UST	Ustekinumab
VDZ	Vedolizumab
